# Metastatic Uterine Sarcomas: A Retrospective Analysis on Clinical Outcomes and Biology

**DOI:** 10.7759/cureus.113605

**Published:** 2026-07-29

**Authors:** Neha Singh, Ankita Rungta Kapoor, Roma Jethani, Anuj Gupta, Zachariah Chowdhury, Akhil Kapoor, Bal Krishna Mishra, Ipsita Dhal, Kunal Ranjan Vinayak, Bipinesh Sansar

**Affiliations:** 1 Pathology, Institute of Medical Sciences, Banaras Hindu University, Varanasi, IND; 2 Radiation Oncology, Mahamana Pandit Madan Mohan Malaviya Cancer Centre and Homi Bhabha Cancer Hospital, Tata Memorial Centre, Homi Bhabha National Institute, Varanasi, IND; 3 Gynecological Oncology, Mahamana Pandit Madan Mohan Malaviya Cancer Centre and Homi Bhabha Cancer Hospital, Tata Memorial Centre, Homi Bhabha National Institute, Varanasi, IND; 4 Medical Oncology, Mahamana Pandit Madan Mohan Malaviya Cancer Centre and Homi Bhabha Cancer Hospital, Tata Memorial Centre, Homi Bhabha National Institute, Varanasi, IND; 5 Oncopathology, Mahamana Pandit Madan Mohan Malaviya Cancer Centre and Homi Bhabha Cancer Hospital, Tata Memorial Centre, Homi Bhabha National Institute, Varanasi, IND; 6 Palliative Medicine, Mahamana Pandit Madan Mohan Malaviya Cancer Centre and Homi Bhabha Cancer Hospital, Tata Memorial Centre, Homi Bhabha National Institute, Varanasi, IND

**Keywords:** endometrial stromal sarcoma, ifosfamide-based treatment, molecular subtyping, uterine leiomyosarcoma, uterine sarcomas

## Abstract

Introduction

Metastatic uterine sarcomas are aggressive malignancies with poor outcomes and a scarcity of real-world data.

Methods

This retrospective study collected data from patients treated over eight years, from January 2018 to December 2025. The included patients had histopathologically confirmed sarcoma arising from the uterus who were deemed unresectable or were metastatic and treated with palliative intent.

Results

Twenty-eight patients were included in the final analysis, and 23 received first-line treatment. High-grade endometrial stromal sarcomas (HG-ESS) and uterine leiomyosarcomas (ULMS) were the most common histologies. The most common metastatic sites were the pelvis/peritoneum (60.7%) and lungs (57.1%). Anthracycline-based regimens, with or without ifosfamide and gemcitabine, plus docetaxel, were the most common first-line regimens. Disease control rate (DCR) was 13/23 (56.52%). Overall survival (OS) for the whole cohort was five months (95% confidence interval (CI): 0.8-9.2 months). By histologic subtype, HG-ESS had the shortest median OS at four months (95% CI: 1.84-6.12 months), whereas ULMS had the best prognosis, with a median OS of 11 months (95% CI: 2.35-19.65 months). Patients on best supportive care had a median OS of one month, whereas those receiving any treatment had a median OS of 10 months (95% CI: 5.9-14 months) (p<0.001). Patients with a maximum tumor dimension of less than 10 cm had significantly better OS than those with more than 10 cm (10 months (95% CI: 4.8-15.2 months) versus five months (95% CI: 2.6-7.4 months); p=0.031).

Conclusion

The prognosis of metastatic uterine sarcomas remains dismal. Further studies are needed to identify the optimal treatment and sequencing while incorporating molecular classification, especially for HG-ESS.

## Introduction

Uterine sarcomas represent rare malignancies of the endometrium with scant data in the reported literature. With the available evidence, they are aggressive and have poor outcomes. Appropriate surgery, as per the stage, is considered the standard of care for early-stage uterine sarcoma when deemed operable. Adjuvant chemotherapy and adjuvant radiotherapy can be considered on a case-by-case basis based on pathological findings [1-3].

The major types of uterine sarcoma include high-grade endometrial stromal sarcoma (HG-ESS), low-grade endometrial stromal sarcoma (LG-ESS), uterine leiomyosarcoma (ULMS), undifferentiated/pleomorphic sarcoma, and adenosarcoma. Apart from these, other rare types of sarcoma can sometimes arise from the endometrium and constitute the remainder of the cases [4,5].

The prognosis varies with stage, and five-year overall survival (OS) drops precipitously from stage 1 to stage 4, with five-year OS in stage 4 reported to be around 10-15%. The type and grade of sarcoma also affect prognosis, with lower-grade sarcomas like LG-ESS having significantly higher five-year OS. Other known prognostic factors like age, menopausal status, and maximum size of tumor have not been studied in detail in the category of patients with unresectable/metastatic uterine sarcoma. The treatment in these patients has been largely derived from the pivotal studies of metastatic sarcoma. Furthermore, due to the rarity of sarcoma and poor accrual, most of these studies have reported combined results across a basket of sarcoma histologies [6-8].

Since response rates and outcomes with different treatment strategies in real-world practice for unresectable/metastatic uterine sarcoma remain unreported, we have attempted to analyze them and compare them with the available evidence.

## Materials and methods

This is a retrospective study conducted at Mahamana Pandit Madan Mohan Malaviya Cancer Centre in Varanasi, India, with data collected from patients treated between January 2018 and December 2025. All patients with histopathologically confirmed sarcoma arising from the uterus who were deemed unresectable or were metastatic and treated with palliative intent were included. Cases where the origin could not be reasonably considered as arising from the uterus were excluded.

Appropriate clinical history, physical examination findings, and histopathological, radiological, and molecular pathology details were extracted from the electronic medical records database. All details regarding treatment initiation, responses to treatment, sequence of therapy, and outcomes were noted.

OS was defined as the time from diagnosis to death from any cause. Progression-free survival (PFS) was calculated from the initiation of systemic therapy to radiological or clinical progression or death, whichever occurred first; patients without an event were censored at their last recorded follow-up. Responses were assessed after every three cycles with repeat contrast-enhanced computed tomography of the thorax, abdomen, and pelvis and classified according to the Response Evaluation Criteria in Solid Tumors (RECIST) 1.1 criteria. Disease control rate (DCR) was defined as complete response, partial response, or stable disease per RECIST 1.1 [9].

The collected data were tabulated and analyzed using appropriate statistical tests by IBM SPSS Statistics for Windows, Version 30.0 (IBM Corp., Armonk, New York, United States). These tests included median and percentages. Survival estimates were calculated using the Kaplan-Meier method and the log-rank test; p-values of <0.05 were considered statistically significant. Median follow-up duration was calculated by the reverse Kaplan-Meier method.

The study was approved by the Institutional Ethics Committee of Mahamana Pandit Madan Mohan Malaviya Cancer Centre and Homi Bhabha Cancer Hospital (approval number: OIEC/11000737/2024/00002). The ethical principles of the Declaration of Helsinki (2013 revision) were followed, and the study was conducted and reported in accordance with the Strengthening the Reporting of Observational Studies in Epidemiology (STROBE) guidelines.

## Results

**Figure 1:**
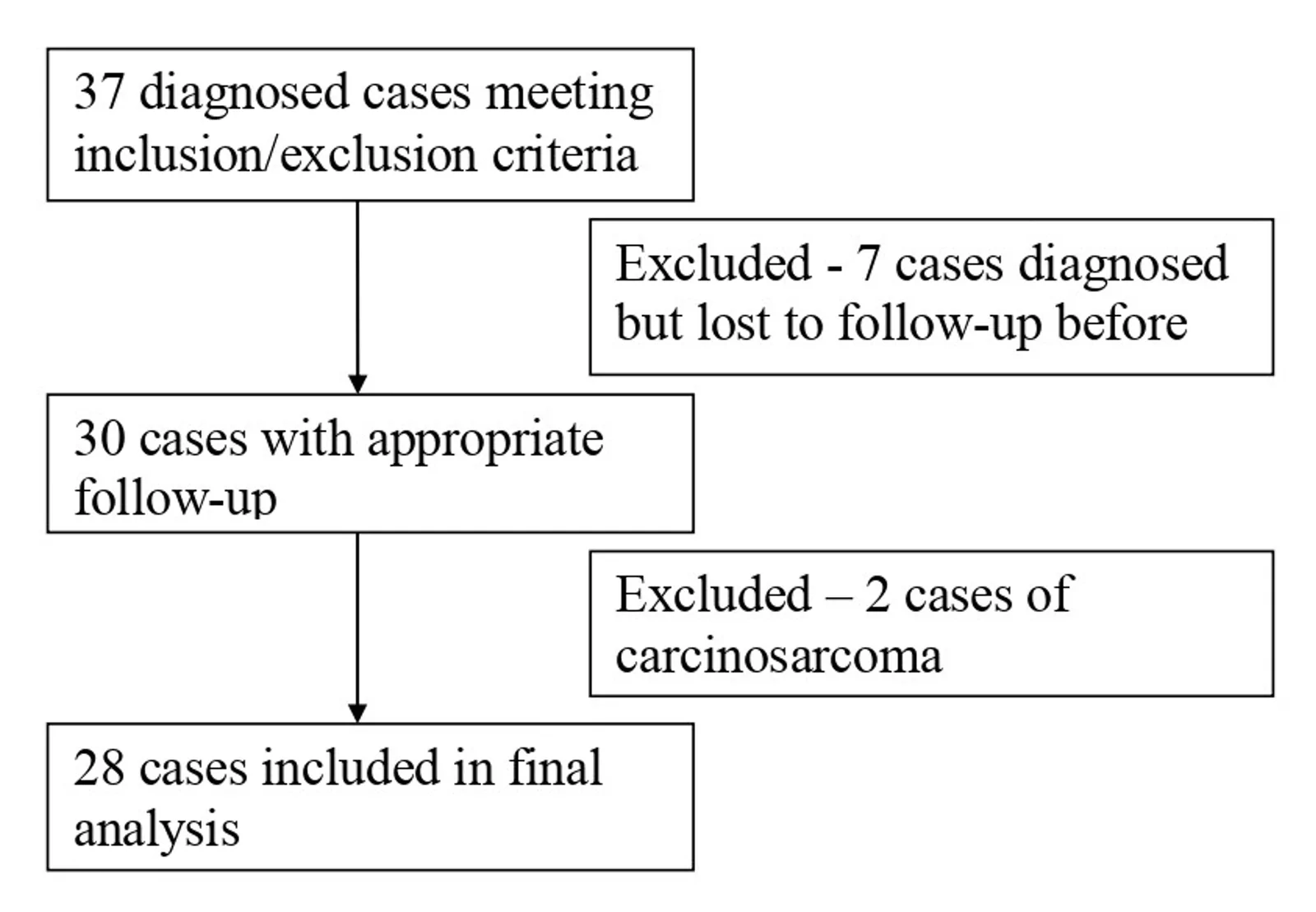
CONSORT diagram of patients included in the study CONSORT: Consolidated Standards of Reporting Trials

A total of 37 patients with histologically confirmed uterine sarcoma who were unresectable/metastatic were identified, of whom 30 patients were found eligible for analysis as shown in the flow diagram (Figure 1).

Two patients initially registered with diagnoses of carcinosarcoma were subsequently excluded following reclassification as non-sarcomatous tumors. The baseline disease and patient-related characteristics are detailed in Table 1.

**Table 1:** Histological subtypes included in the study ESS: endometrial stromal sarcoma

Histologic subtype	Frequency (%)
Leiomyosarcoma	11 (39.3)
ESS: total	13 (46.4)
Low-grade ESS	2 (15.4)
High-grade ESS	11 (84.6)
Ewing sarcoma/Ewing family tumor	2 (7.1)
Rhabdomyosarcoma	1 (3.6)
Pleomorphic sarcoma	1 (3.6)

The histopathological subtype distribution is summarized in Table 2.

**Table 2:** Baseline demographic, clinical, and disease characteristics (N=28)

Age (in years)	
Median (range)	47 (24-85)
<50 years	16 (57%)
≥50 years	12 (43%)
Menopausal status	
Postmenopausal	18 (64.3%)
Premenopausal	10 (35.7%)
Parity	
Median (range)	3 (1-5)
Comorbidities	7 (25%)
Diabetes mellitus	3 (10.7%)
Hypertension	4 (14.3%)
Stage at presentation	
Stage IV	27 (96.4%)
Stage III	1 (3.6%)
Presenting symptoms	
Abdominal pain	16 (57%)
Abdominal distension	7 (25%)
Per-vaginal bleeding	11 (39.3%)
Vaginal discharge	3 (10.7%)
Past history	
History of uterine fibroids	11 (39.3%)
Previous pelvic surgery	19 (67.9%)
Maximum tumor size (in cm)	
Median	10 cm (range: 4-25 cm)
<10 cm	15 (53.6%)
≥10 cm	13 (46.4%)

Estrogen receptor (ER) and progesterone receptor (PR) positivity were observed in 7/13 (53.8%) and 2/9 (22.2%) cases, respectively. ER positivity was seen in 4/10 (40%) of HG-ESS patients and in 2/2 (100%) of LG-ESS patients, respectively. Molecular testing was performed in seven patients (25%), identifying an RB1 loss in one HG-ESS and a TP53 mutation in one rhabdomyosarcoma case. The patient with RB1 loss also had a PDL1 combined proportion score of 55. Three patients with HG-ESS underwent testing for YWHAE rearrangement, and two were positive.

Pulmonary metastases were present in 16/28 patients (57.1%) and pelvic/peritoneal deposits in 17/28 patients (60.7%), respectively. Regional lymph node involvement was seen in 9/28 (32.1%) patients. Eleven patients (39.3%) presented with recurrent disease, with a median disease-free interval of 24 months (range: 4-72; n=11).

Prior adjuvant chemotherapy and radiotherapy had been administered in 3/17 (17.6%) and 2/17 (11.8%) patients, respectively. Three patients with Eastern Cooperative Oncology Group (ECOG) performance status (PS) of 3 and two patients with ECOG PS of 2 were planned for best supportive care. The rest of the patients were planned for systemic treatment, which included 10 patients with ECOG PS of 1 and 13 patients with ECOG PS of 2, respectively. The details of first-line systemic therapy are tabulated in Table 3.

**Table 3:** First-line treatment regimens with response rates DCR: disease control rate; PR: partial response; SD: stable disease; ESS: endometrial stromal sarcoma; VAC: vincristine plus adriamycin plus cyclophosphamide; EWS: Ewing sarcoma

Treatment regimen	Numbers treated (%)	Disease control rate (DCR=PR+SD)	Response rate (PR)
Gemcitabine plus docetaxel	5 (17.9)	3/5 (60%)	3/5 (60%)
Anthracycline±ifosfamide-based	11 (39.3)	6/11 (54.54%)	3/11 (27.27%)
Best supportive care	5 (17.9)	NA	NA
Pazopanib	2 (7.1)	4/7 (57.1%)	3/7 (42.9%)
Hormonal therapy (low-grade ESS)	2 (7.1)		
VAC regimen (EWS)	2 (7.1)		
Gemcitabine alone	1 (3.6)		

Partial response (PR) to first-line therapy was documented in 9/23 (39.1%) patients, while stable disease was documented in 4/23 (17.4%) patients, with a DCR of 13/23 (56.52%). Progression occurred in 10/23 (43.48%) patients. Only 8/23 (35%) patients went on to receive second-line treatment.

By histology, the combined group of other sarcomas (LG-ESS, Ewing sarcoma (EWS)/Ewing family tumor (EFT), rhabdomyosarcoma, and pleomorphic sarcoma) had the highest DCR of 4/5 (80%), followed by ULMS with 6/10 (60%), with HG-ESS having the lowest DCR of 3/8 (37.5%). By treatment regimen, DCR and PR rates have been documented in Table 3.

**Figure 2:**
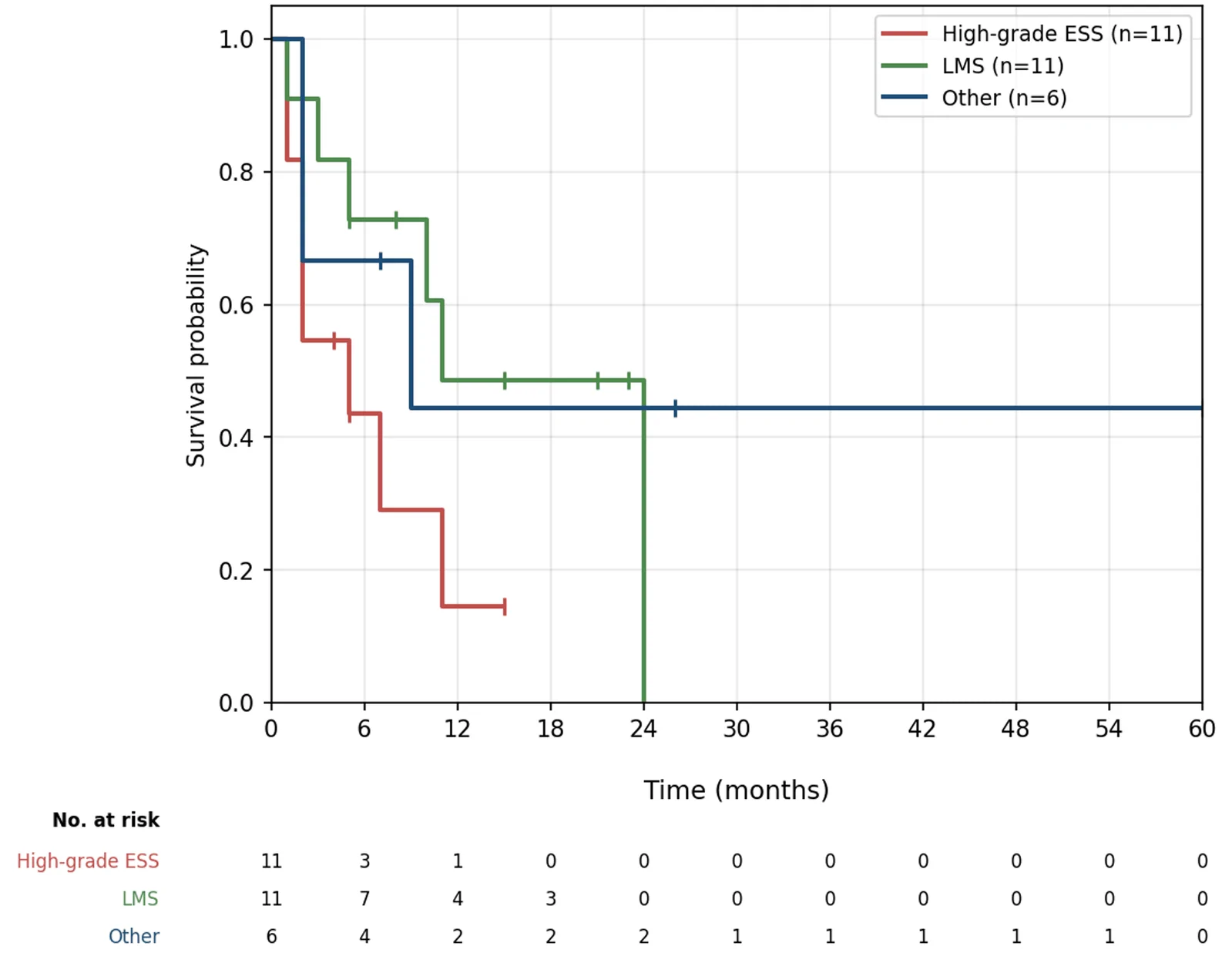
Kaplan-Meier curve for overall survival by histologic subtype (high-grade ESS vs. LMS vs. other sarcomas) ESS: endometrial stromal sarcoma; LMS: leiomyosarcoma

OS for the whole cohort was five months (95% confidence interval (CI): 0.8-9.2 months). By histologic subtype, HG-ESS had the shortest median OS at four months (95% CI: 1.84-6.12 months). ULMS showed the best prognosis with a median OS of 11 months (95% CI: 2.35-19.65 months), while the combined group of other sarcomas had a median OS of nine months (95% CI: 0-22.7 months). This is shown in Figure 2.

The median PFS on first-line treatment was five months (95% CI: 0.7-9.3 months). Patients on best supportive care had a significantly poor median OS compared to those receiving any treatment (one month versus 10 months (95% CI: 5.9-14 months); χ^2^=19.4; p<0.001; log-rank test). Patients with a maximum tumor dimension less than 10 cm had a significantly better OS than the other group (10 months (95% CI: 4.8-15.2 months) versus five months (95% CI: 2.6-7.4 months); χ^2^=4.63; p=0.031; log-rank test). No significant correlation was found between any of the other characteristics mentioned in the results and OS. The median follow-up period was 26 months.

## Discussion

We aimed to evaluate the real-world outcomes of unresectable/metastatic uterine sarcomas. Despite the small sample size due to the rarity and heterogeneity of these cancers, we found some relevant and hypothesis-generating data.

ULMS and HG-ESS were the most common pathological subtypes, similar to other such datasets covering all stages of uterine sarcoma. The lower incidence of LG-ESS could be explained by its good prognosis after initial surgery, leading to lower recurrence rates [7,8].

The high incidence of pelvic surgery history highlights the potential difficulty in the diagnosis of these sarcomas at early stages and missed opportunities for adjuvant treatment, leading to recurrences. In this regard, pathologists may be sensitized to the possibility of sarcomas when noting any atypical presentations. Also, magnetic resonance imaging (MRI) for radiological evaluation may be considered in suspicious cases, given its greater ability to differentiate between benign leiomyomas and malignant leiomyosarcomas [10-12].

The longest median OS was recorded for ULMS, followed by other sarcomas and HG-ESS. The poor prognosis for HG-ESS may be due to its inherently aggressive nature. We also postulate that this could be due to a lack of appropriate randomized studies for this group, with no clear data on the effectiveness of the current first-line therapies used. Most of the data for the treatment of uterine sarcomas has been derived from studies on ULMS. As ULMS shares histology with other non-uterine soft tissue leiomyosarcomas, accrual for phase 2 and phase 3 leiomyosarcoma trials could be completed, leading to more validated treatment strategies for ULMS. This suggests the need for prospective phase 2 studies in HG-ESS to identify the optimal chemotherapy regimens for this subgroup. As HG-ESS is sub-classified based on molecular characteristics, molecular testing should be incorporated and studied for its relation to outcomes and treatment options [13-16].

Among the first-line treatment regimens, the combination of gemcitabine and docetaxel appeared to be the most effective, with higher response rates. However, due to the small number of cases, this can only be considered hypothesis-generating. However, this is supported by several phase 2 studies of this combination, some of which were not randomized. As per the available data, the combination of doxorubicin with trabectedin appears to be the superior alternative, based on a near doubling of median PFS in the LMS-04 trial. However, it included only ULMS patients, and the tolerability of this regimen outside of clinical trials, particularly in patients with ECOG PS of 2, may not be promising [14,15,17,18].

Pazopanib appears to be another option for later-line therapy and first-line treatment in patients considered unfit for chemotherapy. It can be considered in patients with ECOG PS of 2-3, rather than considering best supportive care, as patients without any treatment had a median OS of just one month. Further data are needed for this patient group to guide an optimal strategy. Hormonal therapy in patients with LG-ESS appears to be a reasonable alternative in first-line therapy with similar efficacy and indolent nature as reported in other studies [19,20].

The largest tumor size above 10 cm was found to be significantly associated with poor OS; hence, trials considering the escalation of treatment in this cohort or use of maintenance strategies may enable improvement in outcomes. This is a known poor prognostic factor in early-stage uterine sarcomas, and data for the intensification of adjuvant therapy may also be studied [6].

We hypothesize the small difference between OS and PFS on first-line therapy to be indicative of poor response to second-line treatment and the inability to take second-line therapy due to poor performance status post-progression. The most appropriate option for second-line treatment remains uncertain, with the chemotherapy options not used in first line tailored to the patient's fitness and tolerability profile appearing to be the most suitable choice. For HG-ESS, 40% of patients were ER positive; thus, the option of hormonal therapy can be utilized in a second-line setting or as a maintenance therapy [21-23]. 

The limitations of this study include a small sample size with heterogeneity leading to multiple subgroup analyses, a single-center retrospective study, and a low percentage of molecular testing being performed. 

Targeting BRCA and similar alterations with poly(ADP-ribose) polymerase (PARP) inhibitor therapy is another relevant strategy in the treatment of this group of patients, especially ULMS. This also calls for greater awareness and testing for BRCA alterations similar to ovarian and endometrial cancers [24,25].

## Conclusions

The prognosis of unresectable/metastatic uterine sarcomas remains dismal. ULMS and HG-ESS represent the two major histological categories. While there appears to be reasonable clarity in the treatment paradigm for ULMS, the treatment choices and sequence for HG-ESS need to be better defined through further prospective studies, incorporating molecular subtyping. Other rarer presentations should be treated as per available histology-specific data.
